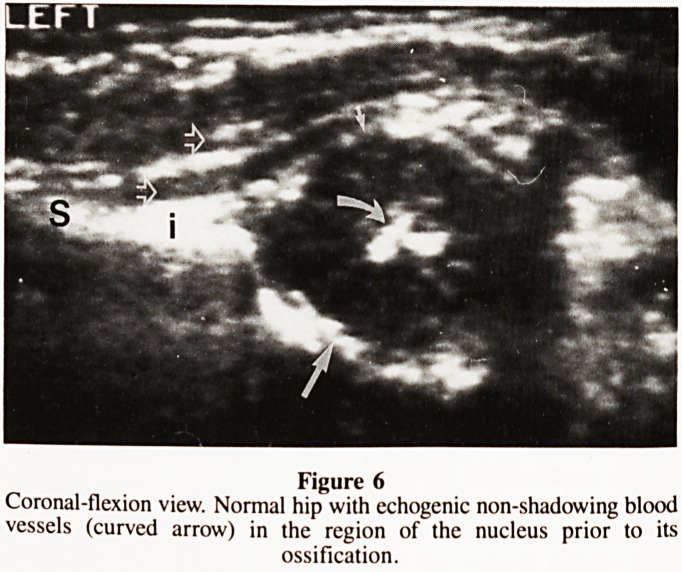# Ultrasound of the Infant Hip

**Published:** 1991-09

**Authors:** Mark Cobby, Nicholas Clarke, Andrew Duncan

**Affiliations:** Departments of Radiology and Orthopaedics, Bristol Royal Hospital for Sick Children St. Michaels's Hill, Bristol; Departments of Radiology and Orthopaedics, Bristol Royal Hospital for Sick Children St. Michaels's Hill, Bristol; Departments of Radiology and Orthopaedics, Bristol Royal Hospital for Sick Children St. Michaels's Hill, Bristol


### Ultrasound of the Infant Hip

**Mark Cobby* MRCP, FRCR, Nicholas Clarke† FRCS, Andrew Duncan FRCR**
Departments of Radiology and Orthopaedics,
Bristol Royal Hospital for Sick Children
St. Michaels's Hill, Bristol.

### INTRODUCTION

Screening of all neonates for congenital dislocation of the hip (CDH) within twenty-four hours of birth has become established clinical practice. The diagnosis of CDH can however, be difficult, and many children with persistent or established dislocations have negative or equivocal signs at birth (Wilkinson, 1985). Since the introduction of screening the incidence of CDH has remained largely unchanged for a number of complex reasons, not all of which are understood (Catford *et al.*, 1982).

CDH encompasses a spectrum of abnormalities ranging from minor dysplasia to frank dislocation. On clinical examination between 15 and 20 infants per 1000 live births will have unstable hips. Most of these resolve without specific treatment with only ten percent going on to eventually dislocate, and a further ten percent showing signs of dysplasia (Standing Medical Advisory Committee DHSS, 1986). Difficulty in knowing which hip will resolve and which requires treatment has led many authorities to advocate immediate splinting of all clinically unstable hips (Dunn *et al.*, 1985). Early splinting in abduction, however, may occasionally result in avascular necrosis of the femoral head. This, and the uncertainty about the natural history of the disease, have resulted in controversy over the detection and optimal management of CDH (Dunn *et al.*, 1985; Leck, 1986; Wilkinson 1985).

Ultrasound has a potentially valuable role in contributing to the diagnosis and management of CDH. It is an accurate and sensitive test for identifying those hips that require treatment (Graf. 1984; Clarke, 1986) and for detecting structural abnormalities in hips that are clinically silent (Berman & Klenerman, 1986; Clarke *et al.*, 1989). It was however, initially received without enthusiasm as it was a difficult and time consuming examination to perform with the static B-scanning equipment on which it was pioneered (Graf, 1980). The development of high quality real time ultrasound has greatly simplified the examination and made it a technique potentially available in all radiology departments.

Ultrasound offers a number of immediate advantages over other imaging investigations. Plain film radiology is relatively unhelpful in the immature pelvis as so little of the hip is ossified. With ultrasound both bony and non-osseous structures are shown allowing the femoral head and its surrounding structures to be demonstrated. It is a dynamic investigation which can be repeated at appropriate intervals to follow the natural history of a questionable hip without submitting, the child to ionizing radiation. The effects of splinting can also be followed to ensure that the femoral head remains appropriately located. If there is failure to maintain concentric reduction, splinting can be abandoned early, so reducing the risk of avascular necrosis (Clarke *et al.*, 1989).

### TECHNIQUE

Most high quality commercially available ultrasound machines are suitable for imaging the infant hip. A 5 MHz near focus transducer is a satisfactory compromise between resolving power and depth of penetration. For children less than three months of age, a 7 MHz transducer will provide higher resolution without undue loss of penetration. A linear array probe is preferred as it is easier to use and has a wider field of view for objects near to the skin than a sector scan.

A combination of two views, using easily identifiable landmarks, are used to demonstrate the anatomical configuration of the acetabulum and detect displacement of the femoral head (Harke *et al.*, 1984; Clarke *et al.*, 1985). The first view is obtained with the hip in the neutral position and the scan section in the transverse or axial plane (transverse-neutral view). The transducer is positioned on the upper femur and advanced cephalad until the femoral head is shown (Fig. 1). The second view is performed with the hip flexed to ninety degrees and the scan section in the coronal plane (coronal-flexion view). The transducer is positioned on the upper femur and advanced posteriorly along the shaft until the femoral head is again shown (Fig. 2).

### ULTRASOUND APPEARANCES

Detailed anatomical correlations of the ultrasound appearances of the hip have been performed and form the basis for interpretation (Yousefzadeh & Ramilo, 1987). Much of the infant hip is composed of hyaline cartilage and appears hypoechoic. The femoral head has a speckled appearance and lies against the echogenic acetabular floor with the triradiate cartilage appearing as an echofree defect (Figs. 1 & 2). The femoral head should have a concentric relationship to the triradiate cartilage in the transverse-neutral position (Fig. 1). Lateral displacement is seen as a gap between the femoral head and the triradiate cartilage whilst superior displacement results in nonvisualization of the acetabulum due to the overlying femoral shaft (Fig. 3).

In the coronal-flexion view the bony acetabulum is surmounted by a triangular cartilaginous roof which extends over the femoral head and to which is attached the triangular labrum (Fig. 2). The labrum is composed of echogenic fibro-cartilage. In the caudal portion of the acetabulum the strongly echogenic band of the legamentum teres can frequently be seen. This view enables the configuration of the acetabulum, the degree of coverage, and the position of the femoral head to be assessed (Figs. 2&4).

The ossific nucleus is visible radiographically between two and six months of age in a girl, and three to seven months in a boy. Ultrasound will demonstrate the nucleus 14 to 21 days earlier but because it casts an acoustic shadow only the lateral portion is shown — the 'half-moon phenomenon' of Graf (Fig. 5). Delayed and asymmetric development of the ossific nucleus is frequently seen in dysplastic hips. Acoustic shadowing from an ossific nucleus greater than 10 mm in diameter will obscure the triradiate cartilage and not allow the exact position of the femoral head to be determined so limiting the value of ultrasound in children more than a year old (Clarke, 1985). Prior to ossification, small blood vessels in the femoral head can be recognized as echogenic, non-shadowing speckles (Fig. 6). Example of normal and pathological hip examinations are illustrated in figures 1 to 6.

### CONCLUSION

Various techniqes for performing ultrasound of the hip have been described. Some of these rely on a single coronal view and complex measurements to classify the hip (Graf, 1984). As with many procedures, familiarity with the range of normal and care in obtaining reproducible standard views, are at least as important as the particular method chosen.

For the moment, ultrasound of the infant hip is confined to assessing questionable abnormalities detected on clinical examination during the first year of life. The dynamic nature of the examination allows the location of the femoral head to be determined in neutral and passive flexion and abduction, as well as under stress using and Barlow and Ortolani manoeuvers. The anatomical configuration of the bony and cartilaginous portions of the acetabulum are also well shown. Serial studies allow close monitoring of the subsequent development of the femoral head and acetabulum in problem hips without submitting the child to ionizing radiation. Ultrasound has the potential of resolving the difficulties concerning the natural history and management of CDH but before it can be used as a screening examination further assessment is required in large controlled trials with adequate long term follow-up (Clarke *et al.*, 1989; Scott, 1989).

### REFERENCES

BERMAN, L., KLENERMAN, L. (1986) Ultrasound screening of hip abnormalities: preliminary findings in 1001 neonates. *British Medical Journal,* **293,** 719-722.

CATFORD, J.C., BENNET, G.C., WILKINSON, J.A. (1982) Congential hip dislocation: an increasing and still uncontrolled disability. *British Medical Journal,* **285,** 1527-1530.

CLARKE, N.M.P., HARCKE, T.H., McHUGH, P., LEE MYUNG SOO., BORNS, P.F., MACEWEN, G.D. (1985) Real-time ultrasound in the diagnosis of congenital dislocation and dysplasia of the hip. *Journal of Bone and Joint Surgery,* **67B,** 406-412.

CLARKE, N.M.P. (1986) Sonographic clarification of the problems of neonatal hip instability. *Journal of Pediatric Orthopaedics,* **6,** 527-532.

CLARKE, N.M.P., CLEGG, J., AL-CHALABI, A.N. (1989) Ultrasound screening of hips at risk for CDH. Failure to reduce the incidence of late cases. *Journal of Bone and Joint Surgery,* **71B,** 9-12.

DUNN, P.M., EVANS, R.E., THEARLE, M.J., GRIFFITHS, H.E.D., WITHEROW, P.J. (1985) Congenital dislocation of the hip: early and late diagnosis and management compared. *Archives of Diseases in Childhood,* **60,** 407-414.

GRAF, R (1980) The diagnosis of congenital hip-joint dislocation by ultrasonic compound treatment. *Archives of Orthopaedic and Traumatic Surgery,* **97,** 117-133.

GRAF, R. (1984) Classification of hip joint dysplasia by means of sonography. *Archives of Orthopaedic and Traumatic Surgery,* **102,** 248-255.

HARCKE, T.H., CLARKE, N.M.P., LEE MYUNG SOO., BORNS P. MACEWEN, D.G. (1984) Examinations of the infant hip with real-time ultrasonography. *Journal of Ultrasound Medicine,* **3,** 131-137.

LECK, I. (1986) An epidemiological assessment of neonatal screening for dislocation of the hip. *Journal of the Royal College of Physicians of London,* **20,** 56-62.

SCOTT, S.T. (1989) Infant hip ultrasound. *Clinical Radiology* **40,** 551-553.

Standing Medical Advisory Committee and the Standing Nursing and Midwifery Advisory Committee for the Secretaries of State for Social Services and for Wales. Screening for the detection of congenital dislocation of the hip (revised 1986). DHSS 1986.

WILKINSON, J.A. (1985) Congenital Displacement of the Hip Joint. Springer-Verlag, Berlin.

YOUSEFZADEH, D.K., RAMILO, J.L. (1987) Normal hip in children: Correlation of US with anatomic and cryomicrotome sections. *Radiology,* **165,** 647-655.

**Figure 1**

Normal transverse-neutral view. The femoral head is centred over the triradiate cartilage and positioned against the bony and cartilaginous acetabulum.

**A**-anterior, **L**-lateral, **p**-psoas major muscle, **1**-echogenic osified portion of pubis, **2**-echogenic ossified portion of ischium, **curved arrow**-cartilaginous femoral head, **open arrow**-greater trochanter, **large straight arrow**-hypoechoic triradiate cartilage with medial through transmission, **arrow head**-hypoechoic pubic carbilaginous portion of the acetabulum, **small arrow**-echogenic ligamentum teres.

**Figure 2**

Normal coronal-flexion view. The femoral head is shown to be contained with a well developed acetabulum.

**S**-superior, **L**-lateral, **i**-ecogenic bony ilium, **curved arrow**-femoral head, **large straight arrow**-acetabulum, **small arrow**-joint capsule, **open-arrows**-gluteus minimus and medius, **1**-hypoechoic cartilaginous acetabular roof.

**Figure 3a**

**Figure 3b**

Posterior superior dislocated hip. The normal relationships of the femoral head to the acetabulum and triradiate cartilage are lost.

**a)** Coronal-flexion view. The femoral head (curved arrow) is located on the ilium (i) The gluteal muscles (open arrow) are heaped up over the femoral head. **S**-supeiro, **L**-lateral.

**b)** Transverse-neutral view. The femoral head (curved arrow) is identified posteriorly but the acetabulum can not be visualized due to the overlying femoral shaft. **A**-anterior, **L**-lateral.

**Figure 4a**

**Figure 4b**

Dysplastic acetabulum with capsular laxity allowing inferolateral subluxation of the femoral head under stress.

**a)** Coronal-flexion view. The femoral head is shown within a dysplastic shallow acetabulum (same key as Figure 2).

**b)** Coronal-flexion view under stress. Inferior and slight lateral displacement of the femoral head is shown.

**Figure 5**

Coronal-flexion view. The femoral head is located within a well developed acetabulum. The ossific nucleus (large crossed arrow) appears as a half-moon casting an acoustic shadow medially (small arrows).

**Figure 6**

Coronal-flexion view. Normal hip with echogenic non-shadowing blood vessels (curved arrow) in the region of the nucleus prior to its ossification.

## Figures and Tables

**Figure 1 f1:**
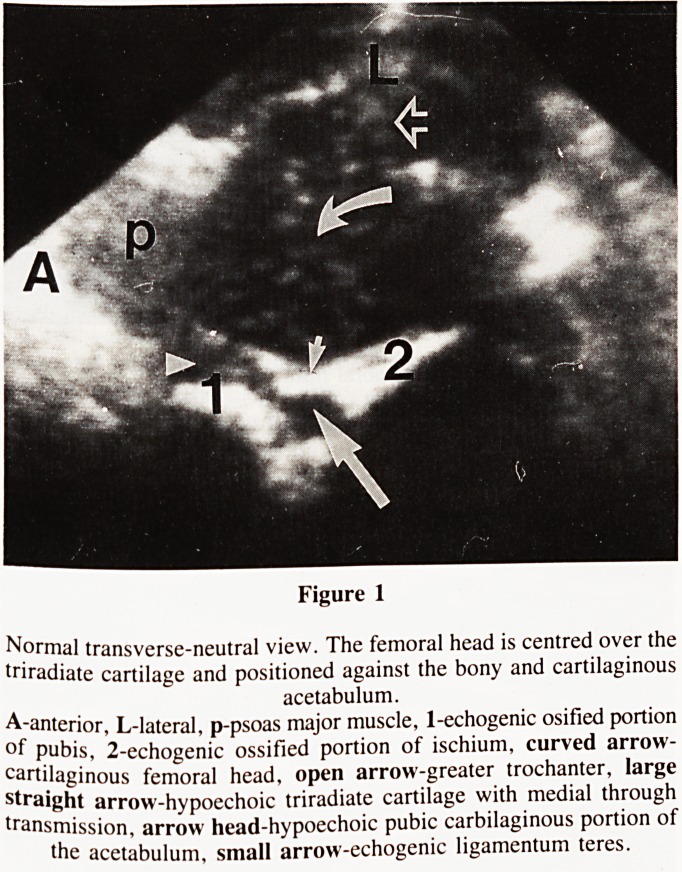


**Figure 2 f2:**
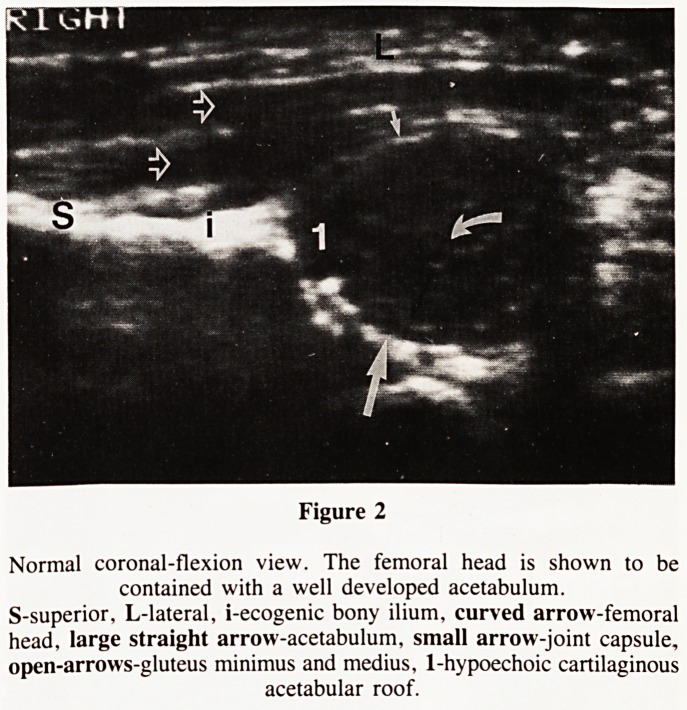


**Figure 3a Figure 3b f3:**
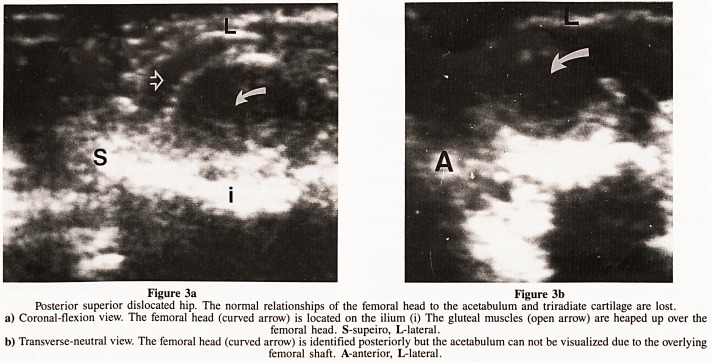


**Figure 4a Figure 4b f4:**
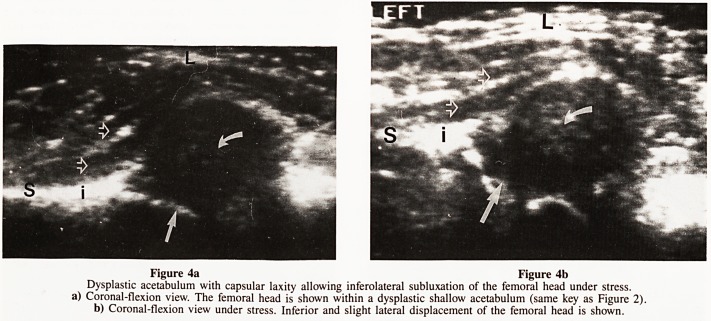


**Figure 5 f5:**
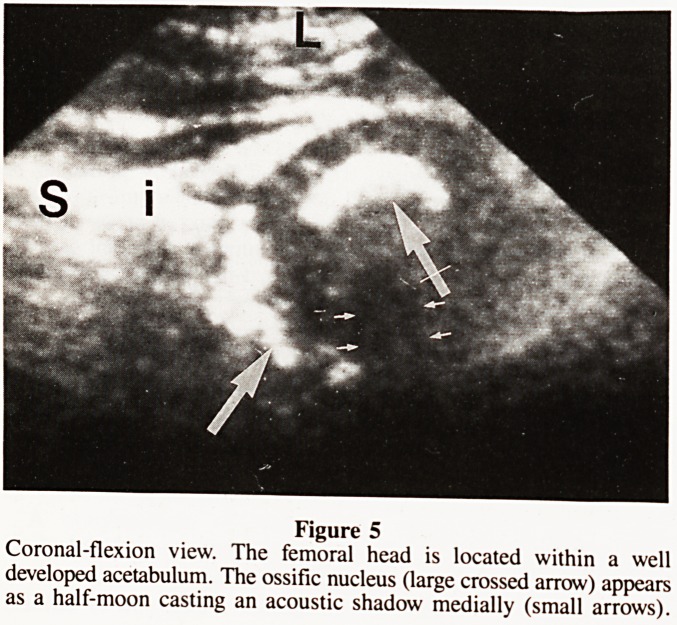


**Figure 6 f6:**